# Comparison of statistical methods and the use of quality control samples for batch effect correction in human transcriptome data

**DOI:** 10.1371/journal.pone.0202947

**Published:** 2018-08-30

**Authors:** Almudena Espín-Pérez, Chris Portier, Marc Chadeau-Hyam, Karin van Veldhoven, Jos C. S. Kleinjans, Theo M. C. M. de Kok

**Affiliations:** 1 Department of Toxicogenomics, Maastricht University, Maastricht, The Netherlands; 2 Department of Epidemiology and Biostatistics, School of Public Health, Imperial College London, London, United Kingdom; California State University Fresno, UNITED STATES

## Abstract

Batch effects are technical sources of variation introduced by the necessity of conducting gene expression analyses on different dates due to the large number of biological samples in population-based studies. The aim of this study is to evaluate the performances of linear mixed models (LMM) and Combat in batch effect removal. We also assessed the utility of adding quality control samples in the study design as technical replicates. In order to do so, we simulated gene expression data by adding “treatment” and batch effects to a real gene expression dataset. The performances of LMM and Combat, with and without quality control samples, are assessed in terms of sensitivity and specificity while correcting for the batch effect using a wide range of effect sizes, statistical noise, sample sizes and level of balanced/unbalanced designs. The simulations showed small differences among LMM and Combat. LMM identifies stronger relationships between big effect sizes and gene expression than Combat, while Combat identifies in general more true and false positives than LMM. However, these small differences can still be relevant depending on the research goal. When any of these methods are applied, quality control samples did not reduce the batch effect, showing no added value for including them in the study design.

## Introduction

Various OMICS techniques are increasingly being used in human population studies to link dietary factors, environmental exposures or therapeutic use of medication to adverse health outcomes and related molecular changes in numerous tissues. Transcriptomics is the study of RNA transcripts produced by the genome using high-throughput technology. Gene expression analysis provides a snapshot of expressed genes and transcripts responding to environmental changes. Since external environmental conditions can change the expression profiling, transcriptomics has become an emerging and promising tool for biomarker discovery [1, 2].

The expression level of mRNAs can be measured using microarray technology. The technique relies on a series of complex chemical reactions between large amounts of RNA molecules and reagents to convert the RNA to cDNA. The gene expression levels are measured by quantifying hybridized and labelled cDNA. The efficiency of these reactions is highly sensitive to experimental settings such as the physical and chemical conditions in which the experiment took place (e.g. temperature, humidity, pH, etc.) and to compliance with the standardized experimental protocol [3, 4]. In real-life studies, numerous biological samples (i.e. hundreds or thousands) from population-based studies are analysed, and the acquisition of gene expression profiles from these biological samples cannot be performed in a single go but rather in lots. The need to perform the analysis on different dates has the potential to induce variability in the measured profiles which is usually referred to as batch effect, producing technical and non-biological variations in the measurements [5–9]. The resulting technically-induced variations in RNA measurements may introduce noise in the data, which in-turn dilutes effects of interest [7]. In order to preserve statistical power and ensure robustness of the identified associations, especially while investigating subtle and complex effects, careful attention should be paid to the methods used to correct for batch effects.

In mRNA microarray technology, the batch effects mainly originate from the isolation of the mRNA, dye labeling of the samples and hybridization onto the microarray [10, 11]. In previous studies, several methods have been applied to account for batch effects that may be present in microarray-based gene expression data sets. These include linear mixed models (LMM) where technical confounders are modelled as random intercepts [10, 12] by assuming a systematic transcript-specific shift in the expression levels in relation to experimental conditions. More complex algorithms such Distance-Weighted Discrimination (DWD) [13], mean-centering Prediction Analysis for Microarrays in R (PAMR) [14, 15], geometric ratio-based methods [16] and Combining Batches of Gene Expression Microarray Data (ComBat) [17] have also been proposed. ComBat, from the Surrogate Variable Analysis (sva) package [18], adjusts batch effects using an empirical Bayesian framework and was shown to outperform the other mentioned methods in a systematic comparison [19]. Quantile normalization in combination with ComBat has been shown to reduce batch effects without dampening the biological effect [6]. On the other hand, it has also been reported that using ComBat for batch effect removal in datasets where groups are distributed among batches in a unbalanced way can hide important associations for both large and small batch sizes [20].

In order to facilitate the quantification of the (possibly differential) measurement error across batches, the inclusion of the same and characterized quality control (QC) sample in all batches is a powerful, but sometimes costly, approach.

In the current study, we propose to investigate the relative performances of the two main approaches to correct for batch effects, linear mixed models correcting for batch as a random effect and Combat. Both approaches will also be investigated in conjunction with the use of QC samples to assess whether these technical replicates, used for calibration purposes, actually improve the models’ performances. For the sake of comparison we also included linear models correcting for batch as a fixed effect. This third approach is equivalent to the genewise one-way ANOVA adjustment performed by some methods like PAMR.

We use existing microarray gene expression data from 251 blood samples of individuals that belong to the EXPOsOMICS project. Standard numerical summaries on the batch effect are estimated from the existing data and used to generate new data. Therefore, we simulate gene expression data using the existing expression data, these numerical summaries and added effect in order to be able to identify the true positives and negatives. We assess the performances of the main approaches to correct for batch effects in the simulated data and the potential added value of including QC samples. Furthermore, the simulated set of effects and batch effects that are introduced in the existing gene expression dataset follow different scenarios, allowing us to evaluate the impact of the effect size, sample size and additional random error. The aim of the simulation study is to assess (i) the impact of batch effect in terms of statistical performances (sensitivity and specificity), (ii) the absolute and relative ability of the proposed methods to improve the models performances in a linear regression context and (iii) the utility of the QCs.

## Methods

### Study Population

The study population is derived from the EU-funded research project EXPOsOMICS which aims to link environmental exposures with biomarkers of exposure, effect and disease. Gene expression levels were obtained from blood samples collected from subjects with asthma and their matched controls (demographics of the population in S1 Table).

### Gene expression data and pre-processing

For each of the study participants, one blood sample was collected at recruitment. Ethical approval was obtained from the Ethics Committee of Basel EKBB and the Ethical Committee of Hospital East Limburg and followed the rules for ethics and data protection, which were in accordance with the Declaration of Helsinki. Written informed consent was given from the subjects. RNAlater was added to the blood samples to preserve RNA quality and the mixture was stored at -80°C within two hours. Total RNA was isolated and hybridized on Agilent 8x60K Whole Human Genome microarrays. Only samples with a 260/280 ratio close to 2 and RNA Integrity Number (RIN) value > 6 were selected for data analysis. All the QCs are the result of one blood withdrawal from one independent subject whose blood was divided into different tubes, mixed with RNAlater and stored using the same procedure as the study samples. Therefore, each of these QCs is a technical replicate. QCs also follow the same quality criteria as the study samples with respect to the 260/280 ratio and RIN values for the RNA isolation. Together with the study samples, two quality control samples (QCs) or technical replicates per batch of microarray hybridization were included in order to assess the potential variation of these QCs which in that case would be a result of the possible variation in the signal across batches.

The original sample set consisted of 291 samples. However, 40 samples belonging to two batches are excluded due to poor quality of the QCs from those batches, resulting in 251 samples and 27 QCs included in the current study. The total number of batches is 14.

### Normalization procedure

Normalization is performed using Bioconductor in R [21]. Local background correction, flagging of bad spots, controls and spots with unacceptably low intensity and log_2_ transformation are applied using the quantile method (github.com/BiGCAT-UM/arrayQC_Module). We adopt two normalization approaches: (i) a two-step approach where independent normalizations of the data from each batch separately are performed followed by normalized data merge, and (ii) a single-step procedure where all samples across batches are normalized together.

After normalization, genes with less than 30% flagged bad spots are selected, transcript replicates are merged by calculating their median and missing values are imputed using the k-nearest neighbors (k-NN, k-value 15) [22] for all samples except for QCs. The total number of probes is 27,522.

### Calibration using Quality Control samples

In some of the analyses, normalization is complemented by applying a multiplicative correction factor to measured gene expression levels and therefore ensuring optimal consistency in measurements obtained across QC samples.

In practice for a given gene *i*, measured in batch *j*, the correction factor *QC*_*ij*_ is defined by the following ratio:

$${QC}_{ij}=\frac{\frac{{\sum_{k=1}^{2}q}_{ijk}}{2}}{\frac{\sum_{j=1}^{b}\sum_{k=1}^{2}q_{ijk}}{n}},$$ (1)

where the numerator is the mean expression level for gene *i* across the k = 2 measurements (there are two QC samples per patch) in batch *j*, and the denominator the mean expression levels of gene i measured across all batches (denoting *b* the number of batches, mean calculated on 2*b values). The resulting set of calibration coefficients are subsequently applied to all gene expression measurements.

### Regression Methods

**Fig 1:**
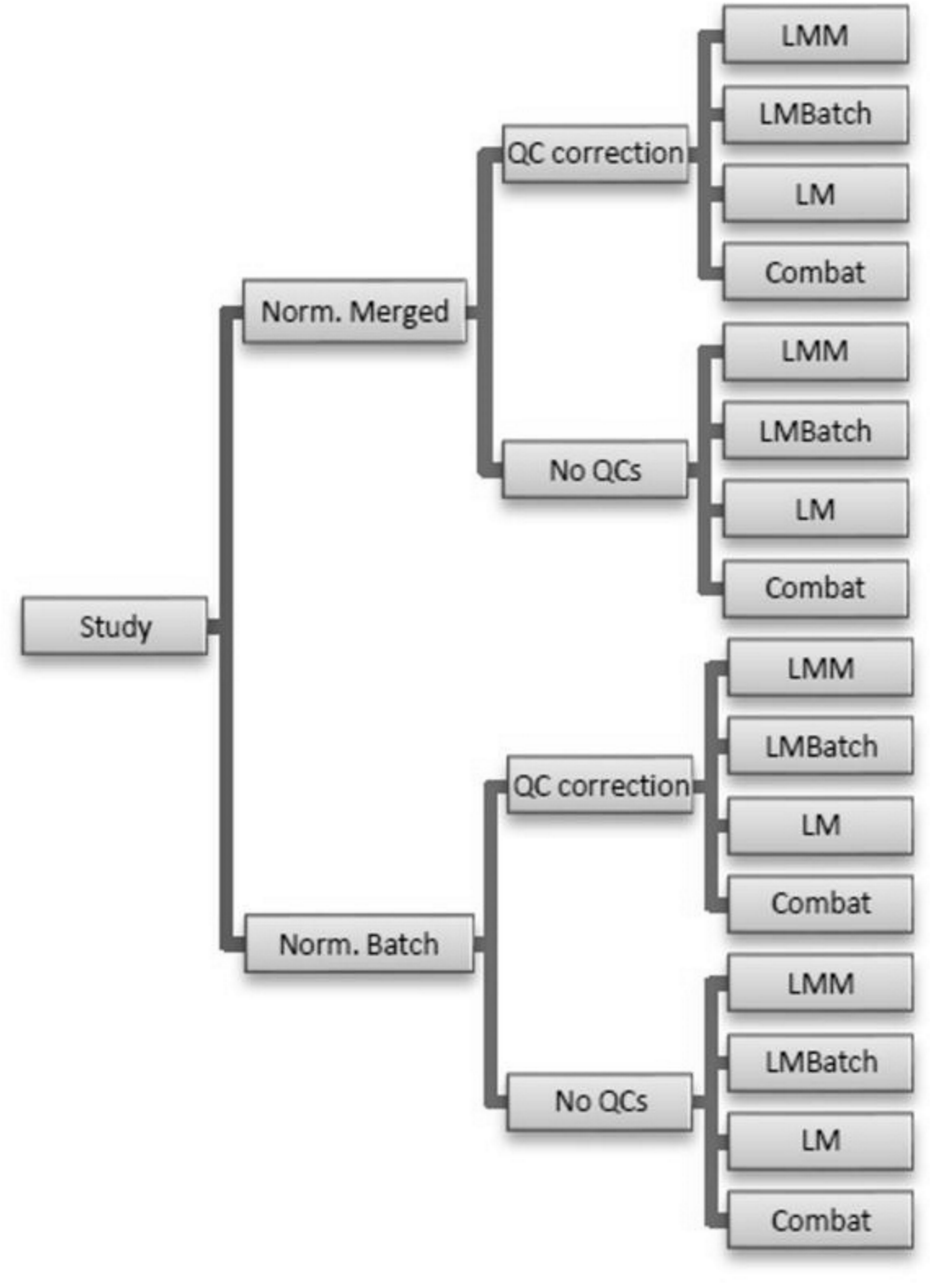
Workflow of the regression methods.

Several analyses are performed for comparison purposes in both normalization per batch and merged normalization. For each normalization method, QC correction either is or is not performed. Batch effects are removed by using maximum likelihood for the linear regression methods and by the empirical Bayesian framework for Combat. The statistical methods that we use are linear mixed models (LMM) correcting for batch as a random effect, linear models (LM) correcting for batch as a fixed effect (LMBatch), LM without batch correction as a control and Combat. Thus, there are two methods of normalization, presence or absence of a QC correction and the four modelling methods for a total of 16 different statistical analyses (Fig 1).

The variables sex and body-mas index (BMI) are used to find association between these variables (one at a time) and gene expression. In Combat a linear regression model using gene expression corrected for batch as the dependent variable is implemented.

### Principal component analysis (PCA)

Principal components analysis (PCA) is used to identify batch effects by examining patterns in plots of the first two principal components.

### De-noising gene expression

The statistical models (Fig 1) are fit to the data and estimated parameters from each model are used to calculate new gene expression data that are corrected for batch effect. PCA plots are generated using the new gene expression data to examine the degree to which the various approaches for removing the batch effect have been successful.

We compute the association between the first five Principal Components (PCs) and the batch variable using R^2^ for the data before and after correcting for batch effect.

### Correlation among QCs

A Pearson correlation test (R package “stats”) is applied to the QC samples after batch effect removal to determine the level of agreement between the QC sample values. If the batch effect removal approach is successful, the QC sample values should be highly correlated.

### Simulation of batch and effect in gene expression

Examining a single dataset across multiple batch correction methods provides limited information for determining how methods compare. This is mainly due to not knowing if there is a batch effect and the actual magnitude of the true effect when it exists. In simulation experiments the random data is generated from a situation where the magnitude of the batch effect is known, which enables the appropriate evaluation of the correctness and suitability of the methods in handling the batch effect. The simulation experiment conducted here creates gene expression data for numerous individuals that are analyzed in batches with two QCs for each batch without the limitation of not knowing the true positives. A known set of effects or “treatment” effects and batch effects is then used to evaluate how well a method procedure corrects for batch while still identifying the treatment effects. Rather than generating random gene expression datasets from normal distributions (an underlying assumption of the analytical methods), data is generated by resampling from a real gene expression dataset. The known set of effects and the batch effect are simulated at random and added to the expression values. The four models are then fit to each randomly-generated dataset. One-thousand (1000) random datasets are generated for each case in the simulation study.

In mathematical terminology, the process is as follows:

The general form of the linear models used in the analyses is

$$Y_{m}=\beta_{0}+X_{1}\beta_{1}+\varepsilon_{m}$$ (2)

where *Y*_*m*_ defines the expression level for sample *m*, *β*_0_ the intercept of the model, *X*_1_ the variable of interest (the effect), *β*_1_ the regression coefficient associated with the variable of interest and *ε*_*m*_ the residual error.

The general form of the linear mixed model used in the analyses has an additional term:

$$Y_{m}=\beta_{0}+X_{1}\beta_{1}+u^{A_{m}}+\varepsilon_{m}$$ (3)

where $u^{A_{m}}$ defines the shift associated with *A*_*m*_, the batch effect variable from sample *m* (10). The rest of the parameters are as defined in the LM equation.

The general form of the model used in the Combat analysis is:

$$Y_{ijm}=\beta_{0_{i}}+X_{1}\beta_{1_{i}}+\gamma_{ij}+\delta_{ij}\varepsilon_{ijm}$$ (4)

where *γ*_*ij*_ and *δ*_*ij*_ represent the additive and multiplicative batch effects of gene *i* from batch *j* [23]. The rest of the parameters are as defined in the LM equation.

Define *X* to represent the array of gene expression responses *X* = {*X*_*1*_, *X*_*2*_, … *X*_*w*_} where *X*_*i*_ is the individual response for a given gene, and define *x* as the realization of that variable from a specific sample, *x* = {*x*_*1*_, *x*_*2*_, … *x*_*w*_}. Define the variable associated with the gene expression data for the quality control as *Q* = {*Q*_*1*_, *Q*_*2*_, … *Q*_*w*_}, with *q* being a realization of *Q* from a given sample. There are 14 batches (*b*) in the dataset we are using to generate data, 251 subjects (*m*) and 27 QCs (*n*). The data for an individual gene for a specific person can then be characterized as *x*_*ijl*_ where *i* refers to the gene, *j* refers to the batch and *l* refers to the individual. Replacing *I* or *j* with a dot denotes an analysis is done over the entire subscript. For example, *x*_*ij•*_ would refer to the values for gene *i* across all individuals in batch *j*. Similarly, define *q*_*ijk*_ where *k* represents each QC in a batch (*k* = {1,2} in this specific study design, except for one batch where there is only one QC available).

Simulation parameters are calculated from the control dataset (EXPOsOMICS) using a deconvolution approach. So, *x* = {*x*_*ijl*_: *i = 1*, …*w*, *j = 1*, …*b*, and *l = 1*, …*m*} and *q* = {*q*_*ijk*_: *i = 1*, …*w*, *j = 1*, …*b*, *k = 1*,*2*} denote the data from the control dataset. The data are first log-transformed and QC corrected. A mean QC value is then calculated across batches:

$$\tau_{i••}=\frac{\sum_{j=1}^{b}\sum_{k=1}^{2}q_{ijk}}{n}$$ (5)

For each gene in the dataset in each batch, a mean gene expression value is calculated after QC correction:

$$q_{ij•}=\frac{{\sum_{k=1}^{2}q}_{ijk}}{2}$$ (6)

$$\mu_{ij•}=\frac{\sum_{l=1}^{m}x_{ijl}-q_{ij•}+\tau_{i••}}{m}$$ (7)

Notice that the term “−*q*_*ij*•_ + *τ*_*i*••_” in (7) is equivalent to the previous equation *QC factor*_*ij*_ in Eq (1) but applied to log_2_-transformed data using a deconvolution approach.

A grand mean for all batches is then calculated:

$$\mu_{i••}^{*}=\frac{\sum_{j=1}^{b}\mu_{ij•}}{b}$$ (8)

Finally, across all batches, a standard deviation for the batch effect is calculated:

$$\gamma =\sqrt{\frac{1}{bw}\sum_{i=1}^{w}\sum_{j=1}^{b}{\left(\mu_{ij•}-\mu_{i••}^{*}\right)}^{2}}$$ (9)

These estimated values are now used to generate new data. The batch correction values are assumed to come from a normal distribution with mean zero and standard deviation*γ* (Eq 9) or N(0, *γ*). For each batch, a random batch effect *r*_*j*_ = rnorm(0, *γ*) is generated, where rnorm is the function for generating normally distributed random numbers in R. The vector *r* = {*r*_*j*_, *j* = 1, …*b*} is a simulated realization of the batch effects for each batch.

In order to simulate “treatment”, a variable is generated with one value per subject and gene. The simulation is implemented assuming that the treatment affected the first 500 genes with value *t*_*i*_ (*i* = 1,2,…,500). The additive effect introduced as treatment is generated as *s*_*ijl*_ = *x*_*ijl*_ + *q*_*ij*•_ − *τ*_*i*••_ + *r*_*j*_ + *t*_*i*_, where "+*q*_*ij*•_ − *τ*_*i*••_" is the QC correction factor, *r*_*j*_ is the randomly-generated batch effect and *t*_*i*_ the additive change in the mean that is expected for gene *i* (note *t*_*i*_ = 0 for i>500). The random treatment, which can be seen as a proxy for an effect of exposure, is generated as rnorm(0,SD) for i≤500 and 0 for i>500, with SD = {0.1, 0.2, 0.3, 0.4, 0.5, 1, 3} for the various simulations. These values are chosen to provide a range of treatment effects from very small to large. The same value is used afterwards as the variable of interest for the regression analysis. Alternatively, simulations without the term "+*q*_*ij*•_ − *τ*_*i*••_" are implemented to compare results with and without QC correction.

Once a dataset is simulated, regression analyses are performed in order to identify genes that are significantly impacted by the treatment. P-values are estimated from the linear mixed models (LMM) approach, linear models (LM), linear models correcting for batch as a covariate (LMBatch) and Combat followed by linear models (LMcom). P-values are adjusted using the Benjamini and Hochberg method with threshold at 5%.

The script is available at:

https://github.com/alespre/Batch_Effect/blob/master/SImulation_batcheffect_QCs.R

The simulations are repeated using an independent experimental dataset (The ENVIRonAGE: ENVIRonmental influence ON AGEing in early life [24]) with a similar sample size to test the statistical methods under different conditions.

Finally, the area under the curve (AUC) is calculated for each simulation in order to quantify the overall performance of the different statistical methods to correct for batch effect (R package “pROC”).

### Random error in the treated data

Random error (*e*_*ijl*_) is added to the simulated expression data such that *s*_*ijl*_ = *x*_*ijl*_ + *q*_*ij*•_ − *τ*_*i*••_ + *r*_*j*_ + *t*_*i*_ + *e*_*ijl*_, where *e*_*ijl*_ = rnorm (0, σ_*ei*••_):

$$\sigma_{ei••}=\sqrt{\frac{1}{m}\sum_{l=1}^{m}{\left(\epsilon_{il•}-\frac{\sum_{l=1}^{m}\epsilon_{il•}}{m}\right)}^{2}}$$

where *ϵ*_*il*•_ is defined as the residuals for gene *i* from subject *l*. Simulations are also run without the QCs correction(*q*_*ij•*_ = 0).

### Reduction of the sample size

The same simulations excluding error (*e*_*ijl*_) are performed with a smaller dataset (first four batches, 85 samples) in order to assess the influence of the population size. Additional simulations were performed to test the effect of the batch size by designing scenarios where the number of samples per batch increases by three (scenario 1 = 14 batches with 3 samples in each batch, scenario 2 = 14 batches with 6 samples in each batch, etc.). This test was run using 100 simulations with SD 0.1 and SD 0.5 as effect sizes in the approach with no quality control samples.

### Unbalanced study design

The same simulations are performed with an unbalanced dataset, meaning that the “treatment” variable is not randomly distributed across batches. The analysis was performed by sorting the metadata values (e.g. “treatment”) in such a way that the first batch contained the highest values, the second batch the second highest, etc. The last batch contained the lowest values. Then, 20% of the samples are randomized, meaning that the variable “treatment” is perfectly sorted according to batch in 80% of the samples and randomly distributed in 20% of the samples.

ANOVA tests are run in order to measure the relationship between batches and”treatment” values. The p-values after sorting the data were very close to zero, while before sorting they were non-significant (before sorting overall non-significant p-values across the simulations as it is expected since samples are randomly distributed among batches). Additional simulations were used to test the effect of the association between batches and “treatment” by designing scenarios where a certain number of samples are randomly distributed and another number of samples are not randomly distributed. Using the sorted data as input, 10% of the samples were randomly distributed among batches, resulting in 90% of the “treatment” variable unbalanced and 10% randomly distributed. This approach was repeated for the next deciles (80% unbalanced and 20% randomly distributed, 70% unbalanced and 30% randomly distributed, etc.). This test was run using 100 simulations with SD 0.5 as effect size in the approach with no quality control samples.

## Results

### Statistical analysis using real variables

S2 Table shows the number of genes with significant treatment effects in the EXPOsOMICS dataset for the statistical methods LMM, LM correcting for batch, LM without batch and Combat followed by LM using sex and BMI as variables of interest. The different statistical approaches gave different numbers of hits. However, the true or false positive rate is unknown.

### PCA for each set of batch correction methods

**Fig 2:**
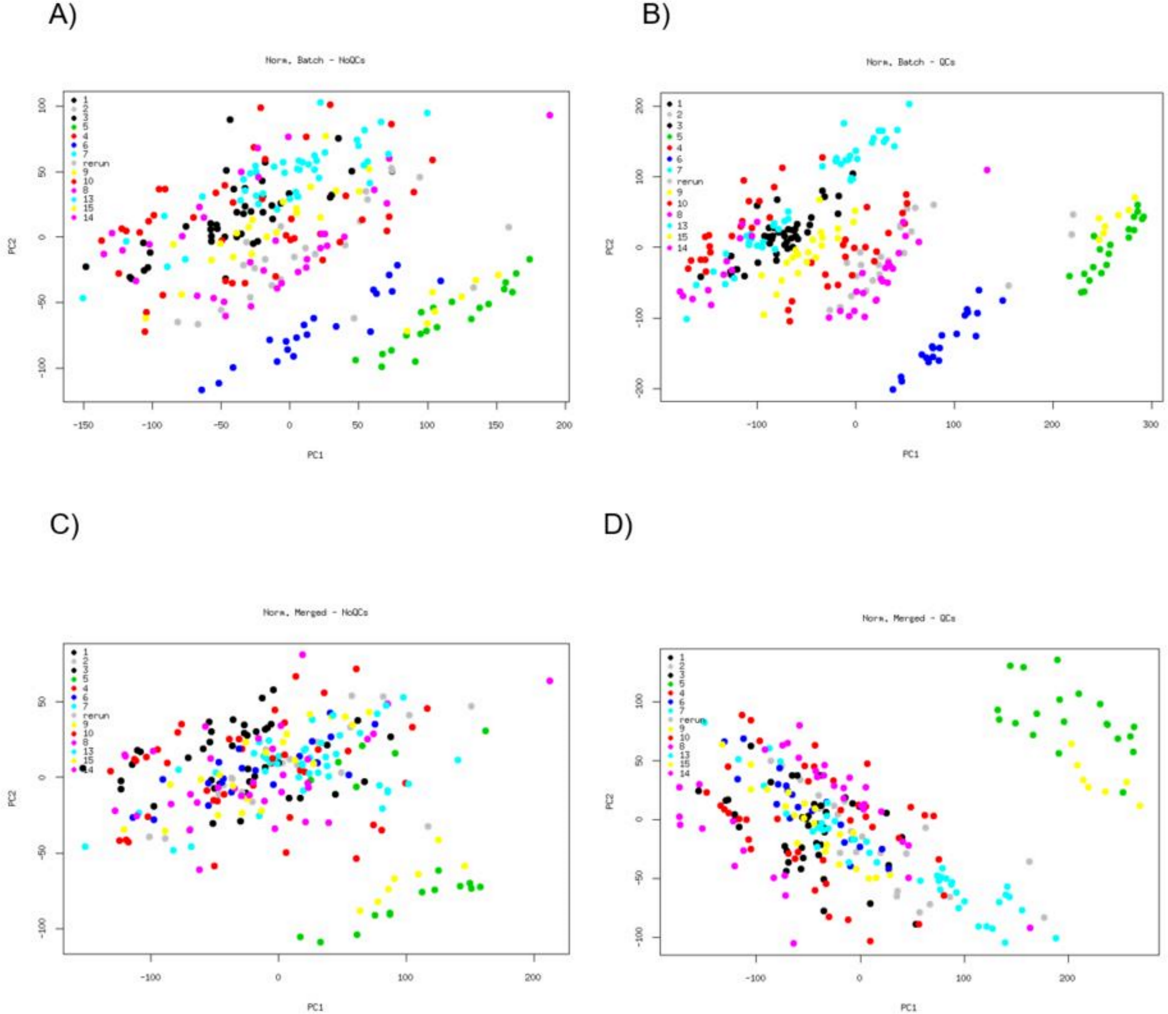
PCA from the four different pre-processing approaches. Each color corresponds to a different batch. A = per batch normalization with no QCs, B = per batch normalization with QC correction, C = merged normalization with no QCs, D = merged normalization with QC correction.

PCA plots of the gene expression data from the four different approaches demonstrate a clear batch effect (Fig 2). The approach that includes per batch normalization and QC factors (B) shows a larger influence of batch than the merged normalization or no inclusion of QCs (A,C and D). Batching could be distinguished to a certain extent in the first Principal Component (PC), the PC that explains the largest proportion of variability in the data. Removing QCs or merged normalization shifts the batch effect to the second PC. The normalization per batch with QCs has the largest amount of variability explained in the PCs (48% for the first PC and 18% for the second PC). The merged normalization with QCs (42% and 9% of the variability) explains the second largest amount of variability, very close to the per batch normalization without QCs (36% and 17%). Merged normalization without QCs (36% and 9%) explains the least.

### De-noising gene expression

PCA plots of the gene expression data from the different approaches after batch effect removal show that the methods efficiently removed the batch effect, even in the cases where batch had a significant impact in the PCA plots from Fig 2 (see S1 Fig).

Table 1 shows the association between the PCs and the batch effect using R^2^ for the pre-processed data and the denoised data after removing batch effect using the three different statistical methods. The correlation values decrease toward zero after correcting for batch effect using any of the three methods, with or without QC correction.

**Table 1:** Association between the first five Principal Components (PC) and the batch effect using R^2^ before and after correcting for batch effect. We underlined the highest correlation value per row.

Normalization	QCs	Method	PC 1	PC 2	PC 3	PC 4	PC 5
Batch	YES	Pre-processed	0.892112	0.766016	0.478084	0.682604	0.242133
Batch	NO	Pre-processed	0.678239	0.278946	0.364995	0.171706	0.373264
Merged	YES	Pre-processed	0.683395	0.772337	0.526378	0.698785	0.864989
Merged	NO	Pre-processed	0.225295	0.317774	0.364029	0.24137	0.290742
Batch	YES	LMM	-0.05030	-0.04602	-0.05376	-0.05324	-0.05176
Batch	NO	LMM	-0.04203	-0.03936	-0.05262	-0.04783	-0.04776
Batch	YES	LMBatch	-0.05479	-0.05089	-0.05477	-0.05474	-0.05398
Batch	NO	LMBatch	-0.05484	-0.05088	-0.05477	-0.05474	-0.05402
Batch	YES	LMcom	-0.04890	-0.05446	-0.05479	-0.05388	-0.05466
Batch	NO	LMcom	-0.03133	-0.05319	-0.05436	-0.05259	-0.05316
Merged	YES	LMM	-0.04843	-0.03939	-0.04990	-0.05247	-0.05004
Merged	NO	LMM	-0.03925	-0.02522	-0.04116	-0.04310	-0.04617
Merged	YES	LMBatch	-0.05469	-0.05096	-0.05461	-0.05481	-0.05399
Merged	NO	LMBatch	-0.05477	-0.05095	-0.05462	-0.05481	-0.05403
Merged	YES	LMcom	-0.04974	-0.05357	-0.05447	-0.05056	-0.05303
Merged	NO	LMcom	-0.04198	-0.04972	-0.05080	-0.04832	-0.04802

### Correlation among QCs

S2 Fig shows the QCs clustering together as expected for repeated samples. All three statistical batch effect removal methods (LMM, LM correcting for batch and Combat) show a high correlation, meaning that there are no extreme disturbances in gene expression after batch effect removal (S3 Table, 6 first rows). For all four methods (LMM, LM correcting for batch, Combat and LM not correcting for batch), the per batch normalization presents the biggest correlation values among QCs but with small differences from the merged normalization. For both per batch and merged normalization, Combat reveals slightly higher QC correlation values. The closer the value is to 1, the less technical effect and disruption is present since a perfect correlation would be expected for repeated samples in the absence of noise. The three methods show differences in magnitude of around 0.002 in the average correlation value. The same correlations are repeated, using a random set of samples from the study instead of QCs, revealing correlation values of around 0.965 (S3 Table, 7–12 rows). There is a difference of 0.02 between the correlation values from the different subjects and the ones from the QC samples, giving an indication of the impact of the inter-individual variation in this dataset. Therefore, the differences between the gene expression correlations among different individuals and from the same individual (QC sample) after batch effect removal have a magnitude 10 times bigger than correlations from the same individual (QC sample) using different batch effect removal statistical methods. Correlations among study samples without batch effect (raw gene expression values from samples from the same batch) show a 10 times higher magnitude than correlations among QCs using different batch effect removal statistical methods (S3 Table, 12–14 rows).

The correlation test might not be enough to capture some systematic changes in gene expression caused by batch since all genes contribute to the test (one test per pair of QCs). Some genes can be more susceptible to batch effects than others [7] and therefore the batch effect should be addressed by a different method.

### Simulation of batch and effect on gene expression

Table 2 summarizes the results from the simulation of 1,000 random datasets. The first column, “Norm”, indicates the type of normalization performed (per batch in all cases). The column “QCs” identifies when quality control sample correction is applied to the gene expression dataset. The next two columns, “Subjects” and “N Batches” provide the population size and the number of batches used in the simulation. “Effect” indicates the simulated treatment magnitude or effect size, expressed as the standard deviation of the normal random values with mean zero. “N effect genes” gives the number of genes in which treatment was added, or in other words, the number of true positives expected to be seen as output from the statistical analysis. The column “Gamma” indicates the standard deviation for the batch effect that was used to generate the random effect (*γ* parameter calculated from the real dataset). “Error” specifies if a random error was added on top of the previous simulated values. The next eight columns show the results of the simulation. Each statistical approach (LMM, LM not correcting for batch, LM correcting for batch and Combat or LMcom) is subdivided into two columns, where the number of expected genes and not expected genes is indicated. Thus, “TP” stands for true positive and “FP” for false positives. The values are calculated from the average of values found from the 1,000 simulations per approach.

**Table 2:** Number of true positives (TP) and false positives (FP) found in the different simulations: with and without QCs for the different effect sizes.

Norm.	QCs	Subjects	N Batches	Effect	N effect genes	Gamma	Error	LMM		LM		LMBatch		Lmcom	
								TP	FP	TP	FP	TP	FP	TP	FP
Batch	No	251	14	3	500	2.72	0	500.0	64.9	500.0	577.4	500.0	64.5	500.0	66.8
Batch	Yes	251	14	3	500	2.72	0	500.0	64.9	500.0	518.8	500.0	64.5	500.0	66.9
Batch	No	251	14	1	500	2.72	0	500.0	64.9	481.3	577.4	500.0	64.5	500.0	66.9
Batch	Yes	251	14	1	500	2.72	0	500.0	64.9	473.8	518.8	500.0	64.5	500.0	66.9
Batch	No	251	14	0.5	500	2.72	0	500.0	64.9	166.6	557.3	500.0	64.5	500.0	67.0
Batch	Yes	251	14	0.5	500	2.72	0	500.0	64.9	143.6	513.2	500.0	64.5	500.0	67.1
Batch	No	251	14	0.4	500	2.72	0	499.5	64.8	83.0	556.7	499.5	64.4	499.7	67.0
Batch	Yes	251	14	0.4	500	2.72	0	499.5	64.9	67.8	512.5	499.5	64.4	499.7	67.0
Batch	No	251	14	0.3	500	2.72	0	494.4	64.3	25.0	556.7	494.4	63.8	495.6	66.4
Batch	Yes	251	14	0.3	500	2.72	0	494.4	64.2	20.3	512.3	494.4	63.8	495.6	66.5
Batch	No	251	14	0.2	500	2.72	0	446.0	60.6	8.2	556.7	445.8	60.1	453.3	63.3
Batch	Yes	251	14	0.2	500	2.72	0	446.0	60.6	7.4	511.5	445.8	60.1	453.2	63.4
Batch	No	251	14	0.1	500	2.72	0	151.9	43.8	7.7	556.8	151.3	43.4	166.6	48.5
Batch	Yes	251	14	0.1	500	2.72	0	151.9	43.8	7.4	511.3	151.3	43.4	166.5	48.6

“Norm” indicates the type of normalization, “QCs” if a quality control sample correction is applied, “Subjects”the population size, “N Batches” the number of batches,“Effect” the magnitude or effect size,“N effect genes” the number of true positives, “Gamma” the batch effect, “Error” if a random error was added. The next eight columns show the number of TP and FP found in the simulations.

Table 2 shows that, for a big effect size (SD 3), all approaches identify the 500 genes. Not correcting for batch (LM) increases the number of false positives to a much larger magnitude than correcting for batch (LMBatch). The smaller the effect size, the fewer true and false positives are identified. In the case of a very small effect size (SD 0.1), the number of true positives is reduced dramatically for all approaches, especially for LM. The three methods that correct for batch effect (LMM, LMBatch and Combat) show similar results. This is also illustrated in the area under the curve values in S5 Table. Small differences are observed in their performance since Combat identifies more true and false positives than the other two approaches. S4 Table shows the average of FDR values for same simulations as Table 2. LMM and LMBatch reveal lower significance levels for large effect size than Combat (1.37E-76 for LMM and 5.97E-77 for LMBatch against 4.67E-64 for Combat).

Very small differences between correcting and not correcting for QCs are observed except for the case of LM where no batch effect removal is applied in the statistical method.

S6 Table demonstrates a similar performance of the statistical methods in an independent experimental dataset. For a big effect size (SD 3), the different approaches identify the 500 genes. The smaller the effect size, the fewer true and false positives are identified.

### Random error in the treated data

In the same way as in Table 2, Table 3 displays the results from the simulation adding random error to the gene expression, treatment and batch effect. Similar trends are observed with respect to the effect size; the number of true positives is reduced more dramatically than in the absence of a random error. The three statistical approaches show very similar results, with Combat showing slightly more true positives and slightly fewer false positives. S7 Table shows lower significance levels for LMM and larger effect size but to a lesser extent than S4 Table.

**Table 3:** Number of TP and FP found in the different simulations after adding random error to the original simulation.

Norm	QCs	Subjects	N Batches	Effect	N effect genes	Gamma	Error	LMM		LM		LMBatch		LMcom	
								TP	FP	TP	FP	TP	FP	TP	FP
Batch	No	251	14	3	500	2.72	Residuals	500.0	33.2	500.0	441.3	500.0	33.2	500.0	31.6
Batch	Yes	251	14	3	500	2.72	Residuals	500.0	33.3	500.0	433.9	500.0	33.2	500.0	31.6
Batch	No	251	14	1	500	2.72	Residuals	500.0	33.2	476.0	441.0	500.0	33.2	500.0	31.6
Batch	Yes	251	14	1	500	2.72	Residuals	500.0	33.3	468.1	433.4	500.0	33.2	500.0	31.7
Batch	No	251	14	0.5	500	2.72	Residuals	498.3	33.0	135.0	439.3	498.3	33.0	498.4	31.5
Batch	Yes	251	14	0.5	500	2.72	Residuals	498.3	33.1	117.9	430.7	498.3	33.0	498.4	31.6
Batch	No	251	14	0.4	500	2.72	Residuals	491.4	32.5	58.6	439.3	491.3	32.4	491.5	31.0
Batch	Yes	251	14	0.4	500	2.72	Residuals	491.4	32.5	48.4	430.7	491.3	32.4	491.5	31.0
Batch	No	251	14	0.3	500	2.72	Residuals	457.5	30.3	18.8	439.3	457.3	30.2	458.4	29.0
Batch	Yes	251	14	0.3	500	2.72	Residuals	457.5	30.4	16.2	430.6	457.3	30.2	458.4	29.0
Batch	No	251	14	0.2	500	2.72	Residuals	327.0	23.0	6.1	439.2	326.8	22.9	329.5	21.9
Batch	Yes	251	14	0.2	500	2.72	Residuals	327.1	23.0	5.4	430.0	326.8	22.9	329.5	21.9
Batch	No	251	14	0.1	500	2.72	Residuals	32.2	5.9	5.6	439.3	32.1	5.9	32.9	5.1
Batch	Yes	251	14	0.1	500	2.72	Residuals	32.2	5.9	5.5	430.1	32.1	5.9	32.9	5.1

Again very small differences are observed between simulations with and without QC correction.

### Reduction of the sample size

Table 4 shows the same as Table 2 but reducing the dataset to 4 batches instead of 14. Trends are similar in terms of effect size; fewer true positives and more false negatives are found in general than in the full dataset. The simulation with a small effect size (SD 0.1) suffers a dramatic loss of true positives, indicating the lack of statistical power to identify weak associations. For small SD values, Combat increases slightly the number of true positives while also increasing the number of false positives (around 1 true positive in exchange for 10 false positives). S8 Table shows slightly lower significance levels for LMM and LMBatch than Combat.

**Table 4:** Number of TP and FP found in the different simulations for the reduced dataset derived from the original population.

Norm.	QCs	Subjects	N Batches	Effect	N effect genes	Gamma	Error	LMM		LM		LMBatch		Lmcom	
								TP	FP	TP	FP	TP	FP	TP	FP
Batch	No	85	4	3	500	2.72	0	500.0	74.5	500.0	487.8	500.0	74.2	500.0	84.0
Batch	Yes	85	4	3	500	2.72	0	500.0	74.4	500.0	475.1	500.0	74.2	500.0	84.3
Batch	No	85	4	1	500	2.72	0	500.0	74.5	317.4	485.8	500.0	74.2	500.0	84.0
Batch	Yes	85	4	1	500	2.72	0	500.0	74.4	310.1	473.8	500.0	74.2	500.0	84.4
Batch	No	85	4	0.5	500	2.72	0	490.2	73.7	77.8	484.9	490.2	73.4	490.5	83.4
Batch	Yes	85	4	0.5	500	2.72	0	490.2	73.6	74.0	473.1	490.2	73.4	490.5	83.7
Batch	No	85	4	0.4	500	2.72	0	467.5	72.4	44.0	484.8	467.4	72.0	468.5	82.1
Batch	Yes	85	4	0.4	500	2.72	0	467.5	72.3	41.8	472.9	467.4	72.0	468.4	82.4
Batch	No	85	4	0.3	500	2.72	0	396.3	69.2	20.6	484.7	396.1	69.0	397.9	78.9
Batch	Yes	85	4	0.3	500	2.72	0	396.3	69.1	19.7	472.8	396.1	69.0	397.8	79.3
Batch	No	85	4	0.2	500	2.72	0	208.3	61.5	8.3	484.6	207.8	61.2	210.2	71.4
Batch	Yes	85	4	0.2	500	2.72	0	208.3	61.4	8.4	472.7	207.8	61.2	210.1	71.8
Batch	No	85	4	0.1	500	2.72	0	7.7	51.0	8.1	485.0	7.6	50.7	8.2	60.4
Batch	Yes	85	4	0.1	500	2.72	0	7.7	50.9	8.0	473.0	7.6	50.7	8.2	61.0

Almost no differences are found between simulations with and without QC correction.

The additional simulations using a varied sample size per batch were run in order to study the performance of the different methods showed a similar performance across methods, where Combat identified slightly more true positives while also identifies more false negatives (S9 and S10 Tables using effect sizes SD 0.1 and SD 0.5, respectively).

### Unbalanced study design

Table 5 shows the same as Table 2 but introducing an unbalanced design in the dataset. Trends are similar in terms of effect size. Fewer true positives with less significance values and more false negatives are found. Combat shows considerably less FP than LMM, although the FDR values of the TP are also considerably less significant than for LMM (S11 Table). Also, there is larger number of FP identified by the LM approach than in a balanced study design.

Almost no differences are found between simulations with and without QC correction.

**Table 5:** Number of TP and FP found in the different simulations for the unbalanced study design dataset.

Norm.	QCs	Subjects	N Batches	Effect	N effect genes	Gamma	Error	LMM		LM		LMBatch		Lmcom	
								TP	FP	TP	FP	TP	FP	TP	FP
Batch	No	251	14	3	500	2.72	0	500.0	79.2	500.0	7383.9	500.0	76.4	499.6	34.8
Batch	Yes	251	14	3	500	2.72	0	500.0	80.3	500.0	7374.6	500.0	76.4	499.5	34.6
Batch	No	251	14	1	500	2.72	0	500.0	79.2	467.6	7366.9	500.0	76.4	485.1	34.9
Batch	Yes	251	14	1	500	2.72	0	500.0	87.0	470.2	6104.1	500.0	83.0	480.9	43.2
Batch	No	251	14	0.5	500	2.72	0	497.9	78.9	235.5	7358.1	497.4	75.9	448.3	35.0
Batch	Yes	251	14	0.5	500	2.72	0	498.0	80.0	239.1	7347.7	497.4	75.9	447.6	34.7
Batch	No	251	14	0.4	500	2.72	0	492.6	78.2	185.3	7357.4	491.7	75.2	429.8	34.9
Batch	Yes	251	14	0.4	500	2.72	0	492.9	79.3	191.3	7343.4	491.7	75.2	429.3	34.7
Batch	No	251	14	0.3	500	2.72	0	471.5	75.7	144.8	7356.8	469.7	72.8	394.1	34.7
Batch	Yes	251	14	0.3	500	2.72	0	472.1	76.9	156.8	7336.9	469.7	72.8	393.6	34.4
Batch	No	251	14	0.2	500	2.72	0	383.6	68.1	127.3	7355.5	381.0	65.3	304.9	33.3
Batch	Yes	251	14	0.2	500	2.72	0	384.5	69.1	138.7	7325.3	381.0	65.3	304.5	33.0
Batch	No	251	14	0.1	500	2.72	0	89.8	36.2	132.2	7533.2	88.5	35.7	66.0	25.6
Batch	Yes	251	14	0.1	500	2.72	0	90.9	37.4	139.6	7633.8	88.5	35.7	65.9	21.4

S12 Table shows the number of true and false positives for the different degrees of association between “treatment” variable and the batch (A) and their mean of the FDR values (B). The column “Random %” shows the percentage of samples that were randomized in the treatment (e.g. 0% corresponds to a perfectly sorted design where all the highest exposures are in batch 1, all second highest exposures in batch 2, etc.). As expected, the more randomized the exposure is across batches, the less p-value in the ANOVA test and the more TP.

## Discussion

In large population studies, batch effects may be introduced as a consequence of sampling procedures or other methodological issues and are likely to be unavoidable due to the need to process and analyse of large numbers of samples. The differences in ‘omics’ signals induced by such methodological factors occurring across batches can be bigger than the influence of the biological variables of interest. Therefore, batch effects may have a large impact on the outcome of studies that are susceptible to this type of noise in the dataset. There are however several methods available to correct or minimize such experimental variation.

We assess the performance of different statistical methods for batch effect removal on gene expression datasets where batch effect and treatment with a range of different effect sizes are simulated. The number of significant hits (FDR<0.05) and their level of significance are extracted from the data analysis of whole-genome gene expression, including both simulated transcripts with added treatment or true positives and transcripts with non-added treatment or false positives. In addition, we evaluate the performance of the statistical methods in two population sizes and the impact of simulated random error, with and without QC correction. The three methods implemented in this study (LM, LMM and Combat) correct efficiently for the introduced batch effects and show similar performance by identifying approximately the same numbers of true and false positives. Nevertheless, small differences in performance are observed depending on the effect size, noise and population size (Tables 2, 3 and 4 and S3, S4 and S5 Tables). S2 Fig presents the potential disturbance of biological signals by removing the batch effect (using the two different methods) as shown by the close clustering of the QCs. It is also shown that differences in the correlations values between different individuals and the same individual (QCs) have a 10 times higher magnitude than the correlation values from the same individual (QCs) using different batch effect removal statistical methods, which suggests a relatively small but potentially still relevant biological impact. These differences among methods were already observed in the data analysis assessing relationships between real gene expression and BMI or sex in S2 Table.

LMM and LM show similar results in our study. LMM is based on the maximum likelihood (ML) and restricted maximum likelihood (REML) methods whereas LM uses the analysis of variance (ANOVA) method. Therefore, LM generates optimal estimators only for balanced designs while LMM generates them for both balanced and unbalanced designs. The need to account for non-independence responses that derive from having different responses by the same batch and therefore adjusting for the covariance structure may differ for each specific dataset. Even though LMM does not outperform LM in this analysis, LMM is in principle able to correct for batch effects in a potentially more efficient way than LM in real datasets in view of its enhanced performance on unbalanced designs.

Although the number of true and false positives from the three statistical methods did not differ to a large extent, the interpretation of the magnitude of the differences between them may depend on the goal of the research studies. For instance, in the development of diagnostic biomarkers, identification of genes for further validation requires high levels of confidence in order to prevent misclassifications which potentially have serious consequences to individuals. On the other hand, the impact of false negatives in a biomarker signature for risk assessment purposes has relatively limited consequences, as the biomarker profile may still identify potential relationships between environmental exposures and biological signals. In general, a stronger identification of true positives using LMM compared with Combat for big effect sizes is observed in this study. Combat identifies in general more true and false positives for small effect sizes, except for the case of added random error where the performance of the different methods is very similar; particularly in cases where there are small sample sizes, Combat shows smaller true positive/false positive rates compared to LMM. An increase of 0.5 true positives in Combat compared to LMM is observed while the increase of false positives is 10, implying a large occurrence of false positives compared to the identification of true positives when batch correction is applied using the Combat method.

On top of the batch effect removal methods available (LM, LMM and Combat), some study designs include QCs (technical replicates of the same sample) for additional batch correction. The variation of signals across batches can be controlled by placing these QCs across the batches and calculating a correction factor (dividing the mean per gene of QC samples from a specific batch by the mean per gene of all QCs) that is applied to the rest of the samples from the same batch. However, this can be costly (e.g. the inclusion of two QCs per batch of 24 samples increases the budget of the array experiment by up to 8%). Our study shows no significant differences in the number of true and false positives between approaches with or without QCs. Therefore, if the inclusion of these additional samples goes at the expense of the number of samples from the actual study population it results in a reduction of the statistical power.

In this study we evaluate the impact of population size, random measurement error, effect size and level of balanced/unbalanced designs on the number of true and false positives from the different statistical methods. In most epidemiological studies, focusing on the link between environmental exposures and gene expression, the magnitude of the associations to be discovered is relatively small. The participants are usually exposed to relatively low doses of environmental factors and therefore the effect sizes are generally rather modest. In addition, sample size is often a limitation in epidemiological studies due to budget restrictions. These two issues increase the likelihood of having noise (e.g. measurement errors or inter-individual variability) mask the potential relationships of interest. The simulations of this study that included low effect size (low SD), added noise and/or decreased population size is expected to generally mimic real environmental studies examples.

The sample size in epidemiological studies should be big enough for the effect of an expected magnitude to become statistically significant. In our study, batch, treatment and error are simulated assuming a Gaussian distribution but this assumption was not implied in the gene expression dataset. This flexibility in the normality assumption leaves open the possibility of assessing these procedures with other types of datasets such as sequencing data. Sources of batch effects from RNA sequencing technology include variability in day-to-day isolation of the mRNA and library preparation, sequencing runs and between different lanes on the flow-cell [5]. In addition, this procedure would be also applicable to other omics datasets (e.g. microRNA, DNA methylation, etc.).

In conclusion, this study shows a comparison of the performance of the most commonly available methods for batch effect removal, LMM correcting for batch as a random effect, LM correcting for batch as a fixed effect and Combat. Small differences among the methods are observed. LMM and LM correcting for batch provide a slightly safer option than Combat by identifying stronger relationships between big effect size and gene expression and better true/false positive rates for small effect size. The study also shows no improvement in the batch correction by adding QCs in the study design when any of the mentioned statistical methods are applied to correct for batch effect.

## Supporting information

S1 Fig **PCA plots after batch effect removal using each of the three methods with and without QCs and using batch normalization (A) or merged normalization (B)**.
(DOCX)

S2 Fig **Hierarchical clustering of the QCs using batch normalization (A) or merged normalization (B)**.
(DOCX)

S1 Table Demographics of the study population. (DOCX)

S2 Table **Number of hits from the variables of interest (sex and BMI) in the EXPOsOMICS dataset after Bonferroni correction (cutoff 0.05) for each of the four models with and without QCs and using batch normalization (A) or merged normalization (B)**.
(DOCX)

S3 Table Correlations of gene expression among QCs and study samples applying different normalization approaches and using the three batch removal methods. (DOCX)

S4 Table Mean of the FDR values from the TP and FP found in the different simulations: with and without QCs for the different effect sizes. (DOCX)

S5 Table Area under the curve (AUC) from the TP and FP found in the different simulations: with and without QCs for the different effect sizes. (DOCX)

S6 Table Mean of the FDR values from the TP and FP found in the different simulations in the independent dataset (The ENVIRonAGE dataset): with and without QCs for the different effect sizes. A) Number of TP and FP found in the different simulations and B) Mean of the FDR values from the TP and FP found in the different simulations.
(DOCX)

S7 Table Mean of the FDR values from the TP and FP found in the different simulations after adding random error to the original simulation. (DOCX)

S8 Table Mean of the FDR values from the TP and FP found in the different simulations for the reduced dataset derived from the original population. (DOCX)

S9 Table **Effect of varied sample size per batch with effect size SD 0.1** A) Number of TP and FP found in the different simulations using different sizes per batch (first column). B) Mean of the FDR values from the TP and FP from the same simulations as A).
(DOCX)

S10 Table **Effect of varied sample size per batch with effect size SD 0.5** A) Number of TP and FP found in the different simulations using different sizes per batch (first column). B) Mean of the FDR values from the TP and FP from the same simulations as A).
(DOCX)

S11 Table Mean of the FDR values from the TP and FP found in the different simulations for the unbalanced study design dataset. (DOCX)

S12 Table A) Number of TP and FP found in the different simulations for the different degrees of association between the “treatment” variable and the batch. B) Mean of the FDR values from the TP and FP found in the different simulations for the different degrees of association between the “treatment” variable and the batch.
(DOCX)
