# Supplementary material for: Comparison of statistical methods and the use of quality control samples for batch effect correction in human transcriptome data
Source: PLoS One. 2018 Aug 30;13(8):e0202947. doi: 10.1371/journal.pone.0202947 (PMC6117018; doi:10.1371/journal.pone.0202947)

S1 Fig. PCA plots after batch effect removal using each of the three methods with and without QCs and using batch normalization (A) or merged normalization (B).

A)


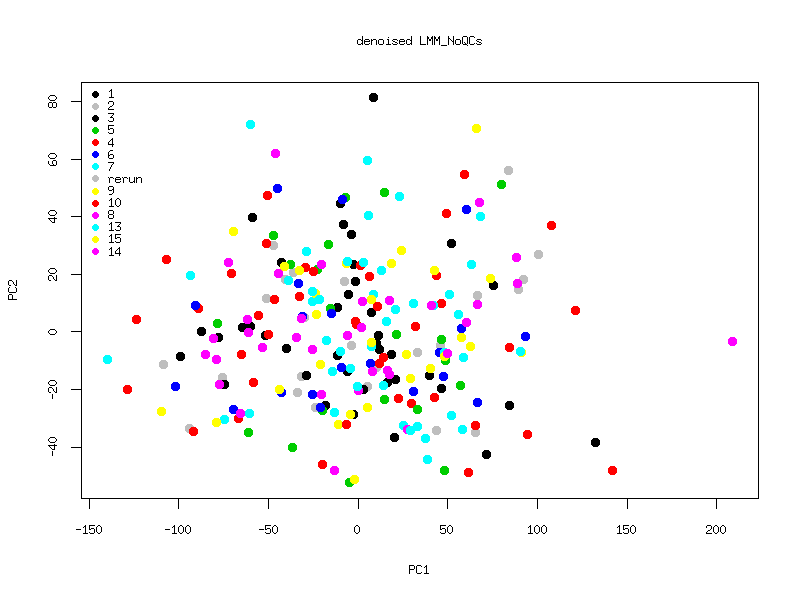

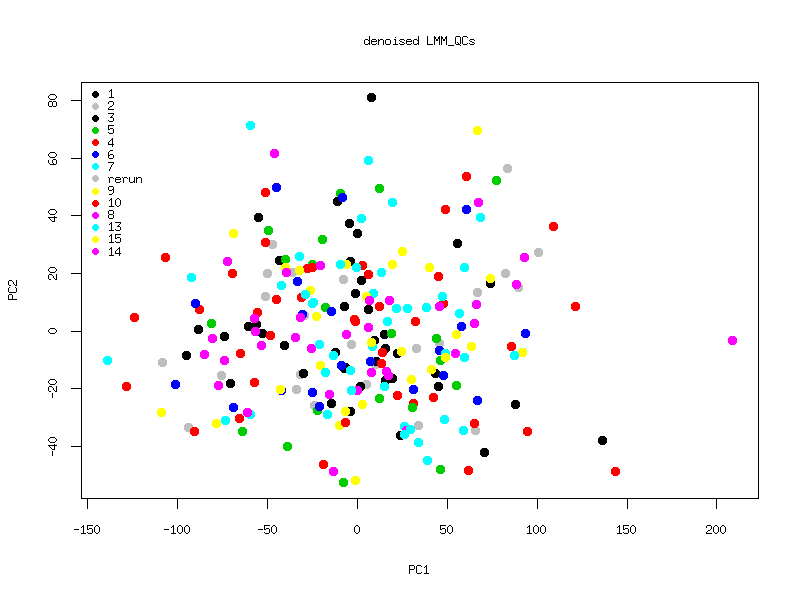

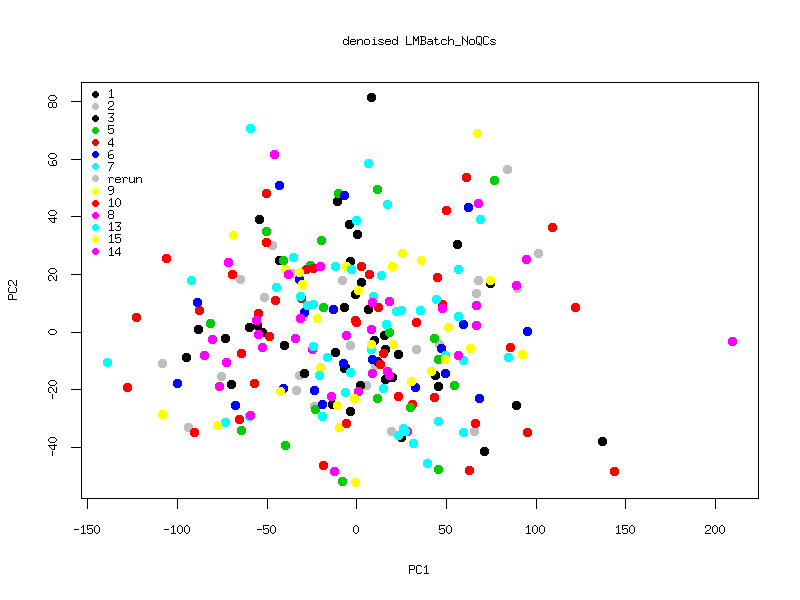

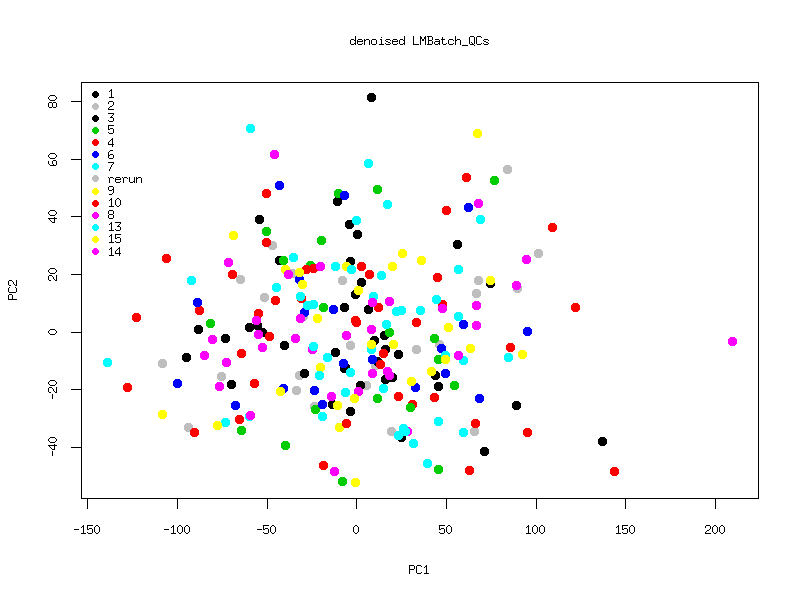


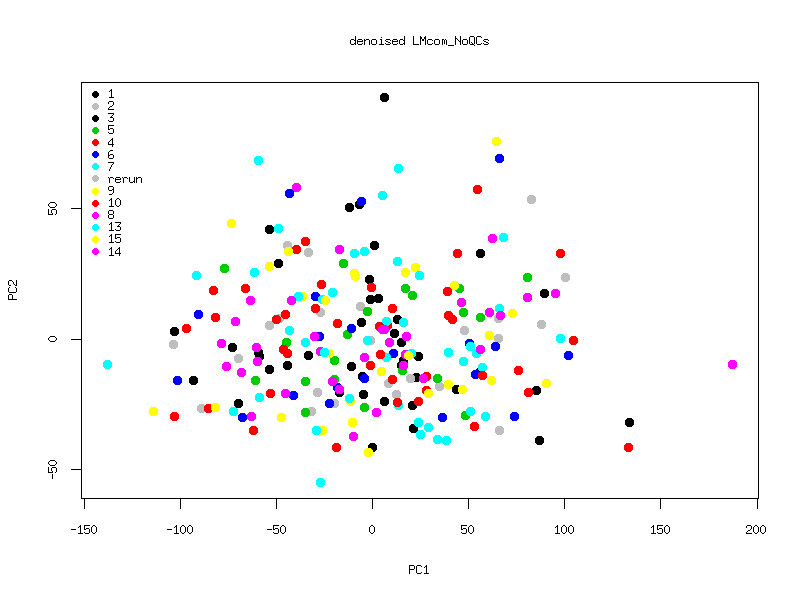

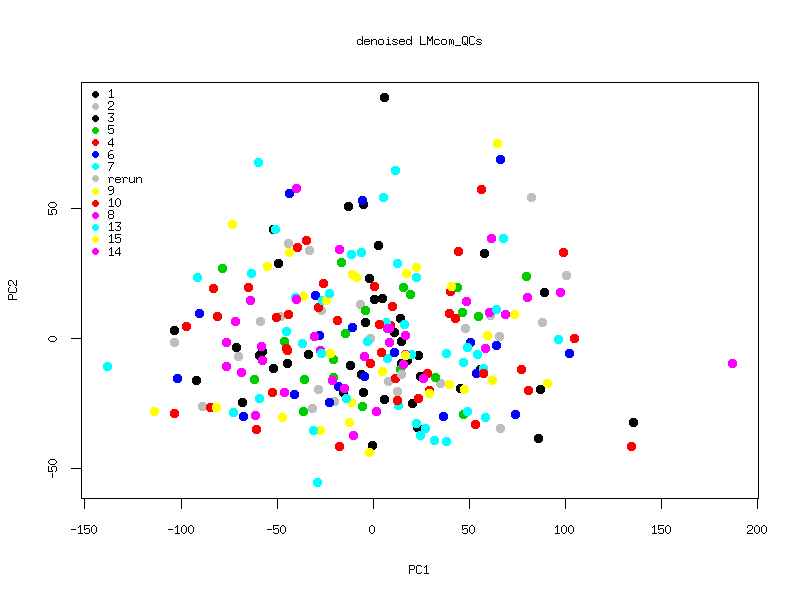


B)


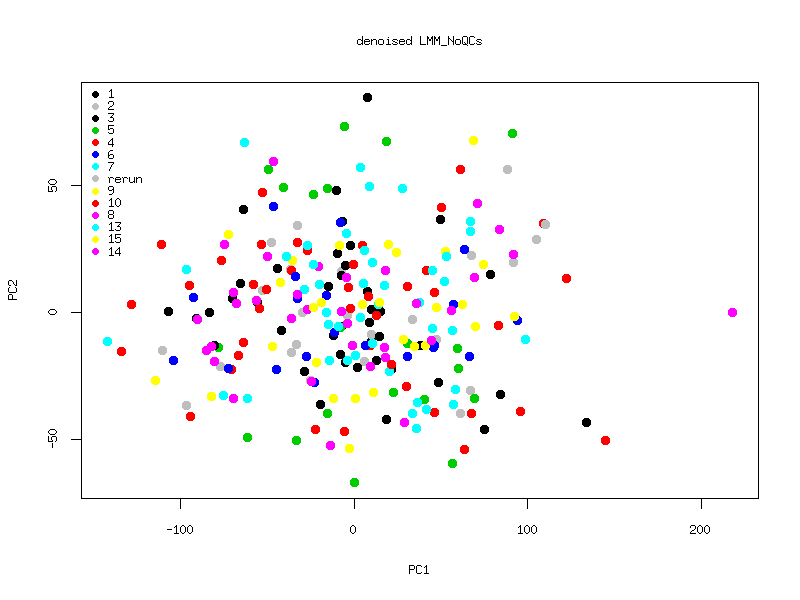

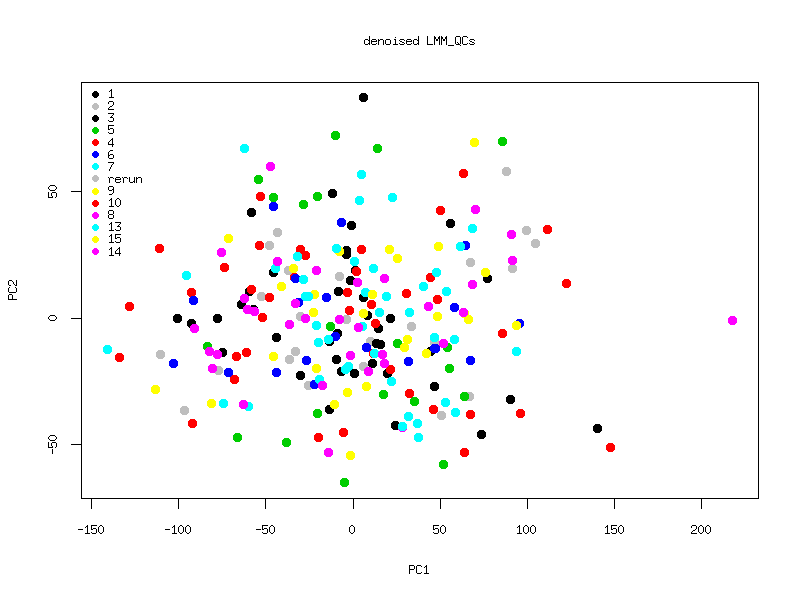


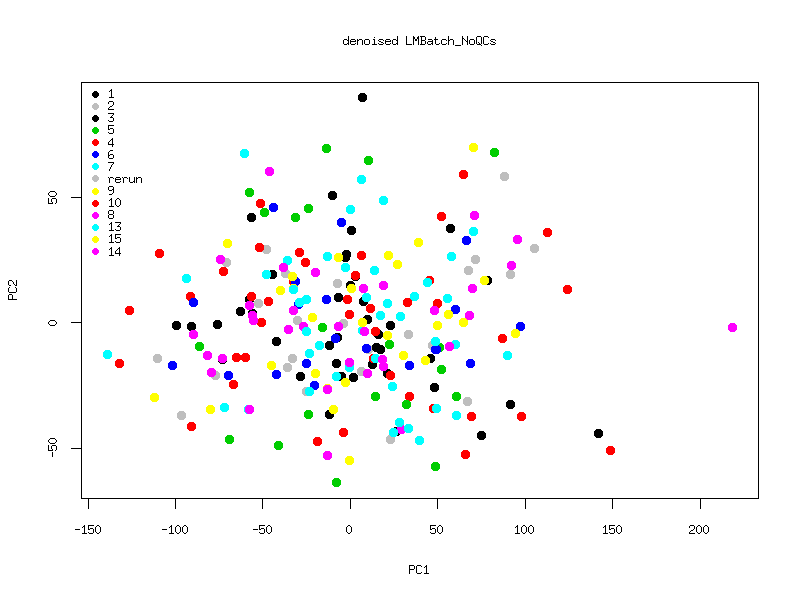

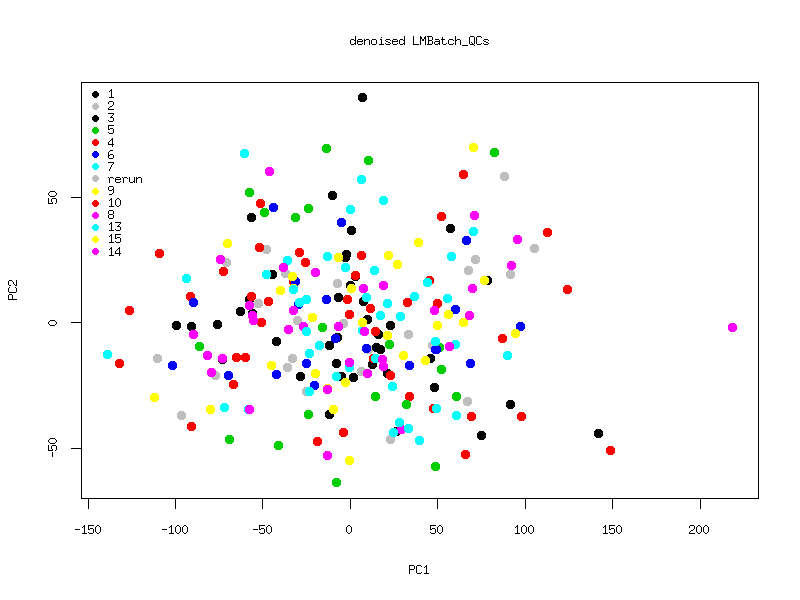


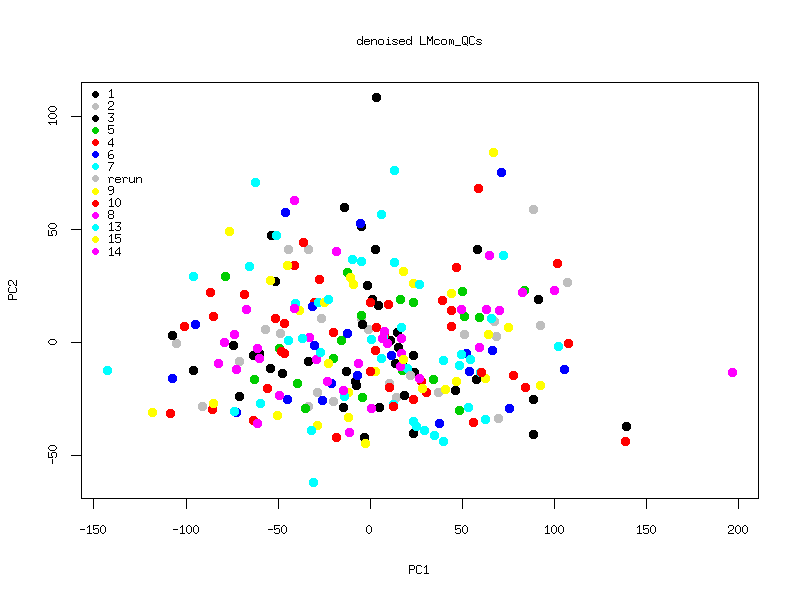

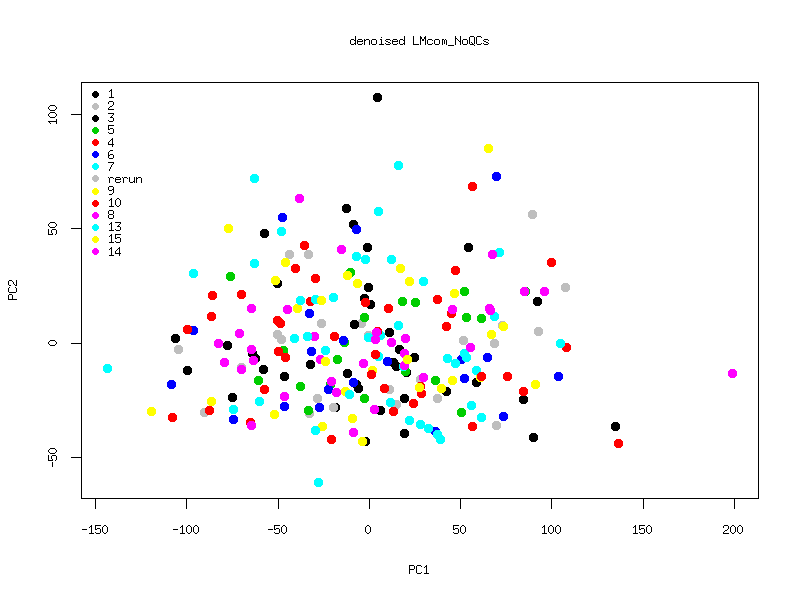

Supplement: S1 Fig — PCA plots after batch effect removal using each of the three methods with and without QCs and using batch normalization (A) or merged normalization (B). (DOCX) [file pone.0202947.s001.docx]
