# Supplementary material for: Comparison of statistical methods and the use of quality control samples for batch effect correction in human transcriptome data
Source: PLoS One. 2018 Aug 30;13(8):e0202947. doi: 10.1371/journal.pone.0202947 (PMC6117018; doi:10.1371/journal.pone.0202947)
Supplement: S2 Fig — Hierarchical clustering of the QCs using batch normalization (A) or merged normalization (B). (DOCX) [file pone.0202947.s002.docx]

S2 Fig. Hierarchical clustering of the QCs using batch normalization (A) or merged normalization (B).

A)

QCs


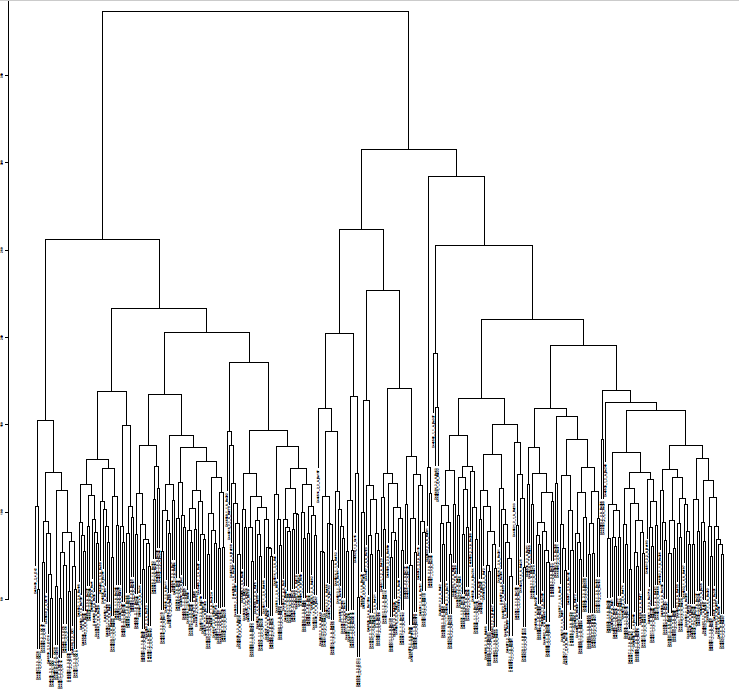


B)


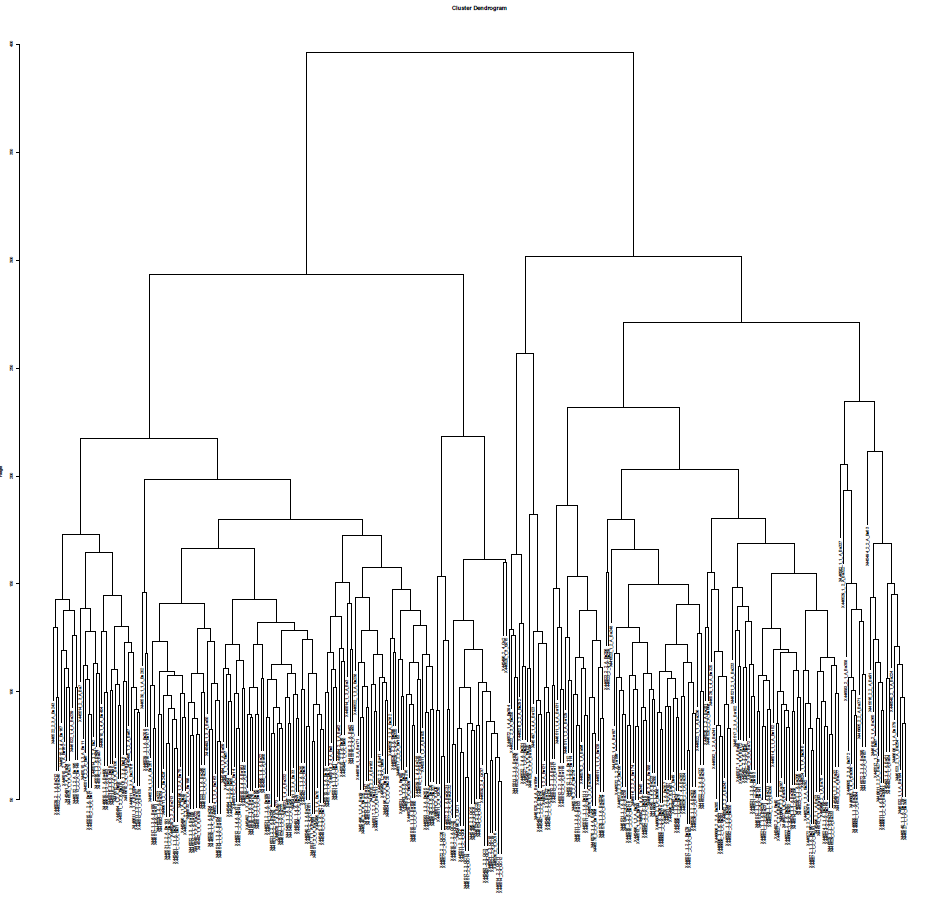


QCs
