# Supplementary material for: Comparison of statistical methods and the use of quality control samples for batch effect correction in human transcriptome data
Source: PLoS One. 2018 Aug 30;13(8):e0202947. doi: 10.1371/journal.pone.0202947 (PMC6117018; doi:10.1371/journal.pone.0202947)
Supplement: S1 Table — (DOCX) [file pone.0202947.s003.docx]

S1 Table. Demographics of the study population.

| BMI and age | | | | |
| --- | --- | --- | --- | --- |
| Variable | N |  | Mean | Std Dev |
| BMI | 406 |  | 25.57 | 4.30 |
| Age | 406 |  | 57.28 | 11.20 |
| Case-control | | | | |
|  | N | % | Cumulative | Cumulative |
|  |  |  | Frequency | Percent |
| case | 204 | 50.25 | 204 | 50.25 |
| control | 202 | 49.75 | 406 | 100 |
| Sex | | | | |
|  | N | % | Cumulative | Cumulative |
|  |  |  | Frequency | Percent |
| Male | 184 | 45.32 | 184 | 45.32 |
| Female | 222 | 54.68 | 406 | 100 |
| Smoking status cumulative | | | | |
|  | N | % | Cumulative | Cumulative |
|  |  |  | Frequency | Percent |
| Never | 265 | 65.27 | 265 | 65.27 |
| Former | 141 | 34.73 | 406 | 100 |
