# Supplementary material for: Comparison of statistical methods and the use of quality control samples for batch effect correction in human transcriptome data
Source: PLoS One. 2018 Aug 30;13(8):e0202947. doi: 10.1371/journal.pone.0202947 (PMC6117018; doi:10.1371/journal.pone.0202947)
Supplement: S2 Table — Number of hits from the variables of interest (sex and BMI) in the EXPOsOMICS dataset after Bonferroni correction (cutoff 0.05) for each of the four models with and without QCs and using batch normalization (A) or merged normalization (B). (DOCX) [file pone.0202947.s004.docx]

S2 Table. Number of hits from the variables of interest (sex and BMI) in the EXPOsOMICS dataset after Bonferroni correction (cutoff 0.05) for each of the four models with and without QCs and using batch normalization (A) or merged normalization (B).

1. B)
