# Supplementary material for: Comparison of statistical methods and the use of quality control samples for batch effect correction in human transcriptome data
Source: PLoS One. 2018 Aug 30;13(8):e0202947. doi: 10.1371/journal.pone.0202947 (PMC6117018; doi:10.1371/journal.pone.0202947)
Supplement: S3 Table — (DOCX) [file pone.0202947.s005.docx]

S3 Table. Correlations of gene expression among QCs and study samples applying different normalization approaches and using the three batch removal methods

* After denoising with the different methods
**Before denoising, no batch effect since all samples belong to the same batch
