# Supplementary material for: Comparison of statistical methods and the use of quality control samples for batch effect correction in human transcriptome data
Source: PLoS One. 2018 Aug 30;13(8):e0202947. doi: 10.1371/journal.pone.0202947 (PMC6117018; doi:10.1371/journal.pone.0202947)
Supplement: S4 Table — (DOCX) [file pone.0202947.s006.docx]

S4 Table. Mean of the FDR values from the TP and FP found in the different simulations: with and without QCs for the different effect sizes

“Norm” indicates the type of normalization, “QCs” if a quality control sample correction is applied, “Subjects”the population size, “N Batches” the number of batches,“Effect” the magnitude or effect size,“N effect genes” the number of true positives , “Gamma” the batch effect, “Error” if a random error was added. The next eight columns show the average in FDR values from the TP and FP found in the simulations.
