# Supplementary material for: Comparison of statistical methods and the use of quality control samples for batch effect correction in human transcriptome data
Source: PLoS One. 2018 Aug 30;13(8):e0202947. doi: 10.1371/journal.pone.0202947 (PMC6117018; doi:10.1371/journal.pone.0202947)
Supplement: S5 Table — (DOCX) [file pone.0202947.s007.docx]

S5 Table. Area under the curve (AUC) from the TP and FP found in the different simulations: with and without QCs for the different effect sizes
