# Supplementary material for: Comparison of statistical methods and the use of quality control samples for batch effect correction in human transcriptome data
Source: PLoS One. 2018 Aug 30;13(8):e0202947. doi: 10.1371/journal.pone.0202947 (PMC6117018; doi:10.1371/journal.pone.0202947)
Supplement: S6 Table — A) Number of TP and FP found in the different simulations and B) Mean of the FDR values from the TP and FP found in the different simulations. (DOCX) [file pone.0202947.s008.docx]

S6 Table. Mean of the FDR values from the TP and FP found in the different simulations in the independent dataset (The ENVIRonAGE dataset): with and without QCs for the different effect sizes. A) Number of TP and FP found in the different simulations and B) Mean of the FDR values from the TP and FP found in the different simulations

A)

B)
