# Supplementary material for: Comparison of statistical methods and the use of quality control samples for batch effect correction in human transcriptome data
Source: PLoS One. 2018 Aug 30;13(8):e0202947. doi: 10.1371/journal.pone.0202947 (PMC6117018; doi:10.1371/journal.pone.0202947)
Supplement: S10 Table — Effect of varied sample size per batch with effect size SD 0.5 A) Number of TP and FP found in the different simulations using different sizes per batch (first column). B) Mean of the FDR values from the TP and FP from the same simulations as A). (DOCX) [file pone.0202947.s012.docx]

S10 Table. Effect of varied sample size per batch with effect size SD 0.5 A) Number of TP and FP found in the different simulations using different sizes per batch (first column). B) Mean of the FDR values from the TP and FP from the same simulations as A)

A)

| N samples | LMM | | LM | | LMBatch | | Lmcom | |
| --- | --- | --- | --- | --- | --- | --- | --- | --- |
| per batch | TP | FP | TP | FP | TP | FP | TP | FP |
| 3 | 428.13 | 38.16 | 10.00 | 997.64 | 417.53 | 22.53 | 395.00 | 38.11 |
| 6 | 490.98 | 77.25 | 8.22 | 509.56 | 490.43 | 72.40 | 491.98 | 92.48 |
| 9 | 498.25 | 121.04 | 18.67 | 526.34 | 498.24 | 119.54 | 498.66 | 137.87 |
| 12 | 499.43 | 84.24 | 57.14 | 1038.33 | 499.43 | 83.68 | 499.57 | 79.20 |
| 15 | 499.77 | 40.10 | 86.67 | 533.36 | 499.76 | 40.11 | 499.84 | 44.64 |
| 18 | 499.92 | 24.05 | 128.21 | 535.05 | 499.91 | 23.88 | 499.92 | 29.46 |
| 21 | 499.95 | 37.02 | 169.98 | 261.51 | 499.95 | 36.61 | 499.97 | 43.93 |
| 24 | 499.95 | 42.97 | 177.16 | 239.43 | 499.95 | 42.82 | 499.98 | 52.63 |

B)

| N samples | LMM | | LM | | LMBatch | | Lmcom | |
| --- | --- | --- | --- | --- | --- | --- | --- | --- |
| per batch | TP | FP | TP | FP | TP | FP | TP | FP |
| 3 | 0.053832 | 0.884735 | 0.781653 | 0.740415 | 0.005337 | 0.495664 | 0.085854 | 0.883846 |
| 6 | 0.005133 | 0.867969 | 0.766961 | 0.74173 | 0.000393 | 0.491894 | 0.004357 | 0.86739 |
| 9 | 0.000865 | 0.88084 | 0.683995 | 0.714804 | 4.81E-05 | 0.494362 | 0.000598 | 0.88014 |
| 12 | 0.000298 | 0.868892 | 0.604005 | 0.703561 | 1.1E-05 | 0.483163 | 0.000225 | 0.869524 |
| 15 | 9.48E-05 | 0.876863 | 0.542249 | 0.684227 | 4.09E-06 | 0.489782 | 7.28E-05 | 0.877665 |
| 18 | 4.42E-05 | 0.86216 | 0.423754 | 0.69024 | 1.46E-06 | 0.4838 | 3.21E-05 | 0.860975 |
| 21 | 1.45E-05 | 0.86237 | 0.342902 | 0.683795 | 2.77E-07 | 0.485152 | 8.02E-06 | 0.858622 |
| 24 | 1.29E-05 | 0.86314 | 0.334361 | 0.680273 | 2.43E-07 | 0.486074 | 6.87E-06 | 0.860011 |
