# Supplementary material for: Comparison of statistical methods and the use of quality control samples for batch effect correction in human transcriptome data
Source: PLoS One. 2018 Aug 30;13(8):e0202947. doi: 10.1371/journal.pone.0202947 (PMC6117018; doi:10.1371/journal.pone.0202947)
Supplement: S11 Table — (DOCX) [file pone.0202947.s013.docx]

S11 Table. Mean of the FDR values from the TP and FP found in the different simulations for the unbalanced study design dataset.
