# Supplementary material for: Comparison of statistical methods and the use of quality control samples for batch effect correction in human transcriptome data
Source: PLoS One. 2018 Aug 30;13(8):e0202947. doi: 10.1371/journal.pone.0202947 (PMC6117018; doi:10.1371/journal.pone.0202947)
Supplement: S12 Table — A) Number of TP and FP found in the different simulations for the different degrees of association between the “treatment” variable and the batch. B) Mean of the FDR values from the TP and FP found in the different simulations for the different degrees of association between the “treatment” variable and the batch. (DOCX) [file pone.0202947.s014.docx]

S12 Table. A) Number of TP and FP found in the different simulations for the different degrees of association between the “treatment” variable and the batch. B) Mean of the FDR values from the TP and FP found in the different simulations for the different degrees of association between the “treatment” variable and the batch.

A)

B)
